# Drug information update. Unconventional treatment strategies for schizophrenia: polypharmacy and heroic dosing

**DOI:** 10.1192/pb.bp.115.053223

**Published:** 2017-06

**Authors:** Bret A. Moore, Debbi A. Morrissette, Jonathan M. Meyer, Stephen M. Stahl

**Affiliations:** 1 U.S. Army Regional Health Command-Central, San Antonio, Texas, USA; 2 Neuroscience Education Institute, Carlsbad, California, USA; 3 California Department of State Hospitals, Sacramento, California, USA; 4 Department of Psychiatry, University of Cambridge, Cambridge, UK

## Abstract

The majority of patients respond to antipsychotic monotherapy at standard doses, but a subset of patients will require more heroic measures that include antipsychotic polypharmacy and high-dose monotherapy. Indeed, research has shown that roughly 30% of patients with psychosis are prescribed multiple antipsychotic medications. We discuss the potential benefits and challenges of these approaches and provide a rationale for why and when they should be utilised.

Schizophrenia is one of the most challenging psychiatric disorders faced by clinicians today, affecting roughly 1% of the population.^1^ Many out-patient clinics and virtually all in-patient facilities manage these often complex clinical cases. Since the introduction of the first-generation antipsychotics (FGA) and subsequent development of the dopamine hypothesis, blockade of dopamine 2 (D_2_) receptors has been the primary goal of pharmacological intervention. Even with the development and proliferation of the second-generation antipsychotics (SGA), D_2_ blockade remains central to effective control of positive symptoms whereas serotonin 2A (5-HT_2A_) plays an important but secondary role in the modulation of dopamine, theoretically improving negative symptoms while reducing the risk of extrapyramidal symptoms.^2^

The term ‘dopamine hypothesis’ of schizophrenia is slowly being phased out, as more sophisticated imaging studies have identified the nature of the dopamine dysfunction and its locus within the striatum. The striatum is a major component of the basal ganglia, and is divided functionally into the dorsal sensorimotor portion, the central associative striatum, and the ventral limbic striatum. Recently it has been found that the best correlation between positive symptoms of psychosis in schizophrenia and levels of D_2_ activity is in the rostral caudate, a portion of the central associative striatum.^3,4^ We thus have *in vivo* confirmation of excessive D_2_ neurotransmission and the relationship to the positive symptoms of schizophrenia. In the limbic striatum low dopamine release is directly related to the severity of negative symptoms; the lower the dopamine levels, the greater the negative symptom severity.^3^

While the goal of treatment is normalisation of D_2_ neurotransmission in the striatum, a subset of patients do not respond to traditional treatment algorithms which include antipsychotic monotherapy at dosing levels defined in registrational trials. Consequently, many patients continue to exhibit uncontrolled positive and negative symptoms as well as manifesting aggressive and impulsive behaviours.^5^ It has been argued that ‘unconventional’ strategies for better management of treatment-resistant psychosis should be employed.^6–8^ The two primary methods we discuss in the following pages include antipsychotic polypharmacy and high-dose monotherapy.

## Why do standard treatments sometimes fail?

To effectively treat the positive symptoms of schizophrenia it is important to achieve at least 60% striatal D_2_ blockade with antipsychotics.^9^ The exception is clozapine, which has been shown to have some antipsychotic effect at as little as 20% blockade.^10^ Beyond 80% D_2_ occupancy, risk of extrapyramidal side-effects (EPS) increases, leading to non-adherence and treatment failure. Therefore, the ‘sweet spot’ of D_2_ receptor blockade for most patients is between 60 and 80%.^6^ However, in some treatment-resistant cases, particularly when aggression and impulsivity are of concern, moving beyond 80% may be necessary.^6,8,11–14^ Moreover, there is significant evidence from the literature that a substantial portion of patients tolerate plasma antipsychotic levels consistent with >80% D_2_ occupancy.

One of the early imaging studies from 1997 noted that a large fraction of patients stabilised on low-dose oral haloperidol had plasma levels associated with >80% D_2_ occupancy;^12^ however, prior clinical studies found that even at a plasma haloperidol level of 6 ng/ml (predicted 90% D_2_ occupancy), only 30% experienced adverse effects.^13^ A similar pattern is seen with risperidone. The pivotal trials studied doses of 10 mg and 16 mg, which correlate with a plasma active moiety level of 70 ng/ml and 112 ng/ml, respectively.^15^ The plasma level of 70 ng/ml would correspond to a D_2_ occupancy of 85%;^14^ however, despite this high level of predicted D_2_ occupancy, the proportion of patients on 10 mg who required anti-Parkinsonian treatment was only 31%.^16^

### Pharmacokinetic failure

Pharmacokinetics is the umbrella term that covers drug absorption, bioavailability, distribution, metabolism and excretion. The role of pharmacokinetics in antipsychotic treatment failure is relatively simple. Through abnormal pharmacokinetic processes such as poor absorption, rapid metabolism and enzymatic polymorphisms, antipsychotic plasma levels do not reach the threshold associated with 60% striatal D_2_ occupancy. This leads to continued psychotic symptoms. It also leads to frustration and confusion for clinicians in that the patient does not sufficiently respond to standard treatment algorithms.

### Pharmacodynamic failure

Pharmacodynamics encompasses receptor binding and sensitivity, postreceptor effects, and chemical communication. Pharmacodynamic treatment failure with regard to antipsychotics is the inability to provide significant amelioration of psychotic symptoms in spite of achieving plasma levels associated with at least 60–80% D_2_ occupancy.^17^ Although adequate drug plasma levels are achieved, patients with treatment-resistant psychosis present with continued positive, cognitive and aggressive symptoms. The treatment failure associated with pharmacodynamic influences is hypothesised to be related to lack of D_2_ receptor sensitivity or hypersensitivity. When patients manifest a lack of extrapyramidal adverse effects or akathisia, increasing drug doses to achieve plasma levels that are associated with >80% D_2_ blockade may be necessary to provide symptom control.^18–20^ The overriding principle is that there are a subset of patients who both tolerate and require high levels of D_2_ antagonism for symptomatic relief.

### The importance of being patient

While recent studies have demonstrated that minimal response after 2 weeks on a particular antipsychotic dose portends a low likelihood of week 6 response on that dose, the full therapeutic effects of adequate D_2_ receptor blockade in schizophrenia may not be apparent until many weeks or months later.^7^ Therefore, patience in pharmacological treatment of psychosis is critical when a patient exhibits partial response.^7,11^ For example, Robinson and colleagues found that in a sample of 118 first-episode patients with schizophrenia or schizoaffective disorder only 20% responded to treatment at 4 weeks. The picture was quite different at 52 weeks; roughly 87% responded to treatment.^21^ Other studies of ziprasidone, risperidone and olanzapine have shown continued improvement over several months of treatment.^11^

## Strategies to use prior to heroic measures

We believe polypharmacy and high dosing should not be the initial approach to treating schizophrenia. However, considering that roughly 30% of patients with psychosis are on multiple antipsychotics, the practice is far from rare.^22,23^ In an effort to address the growing practice of antipsychotic polypharmacy and high dosing of antipsychotics in spite of little support in the literature, Stahl provides 12 case-based recommendations.^7^ We review several below.

### Utilise monotherapy first to include clozapine

Sequential trials of at least two SGAs are recommended. If both trials fail, consideration of an FGA is appropriate. Also, it is important to not overlook clozapine as monotherapy. The efficacy of clozapine in treatment-resistant schizophrenia, particularly with regard to aggression and violence, is well documented.^24–26^ However, some clinicians may be hesitant to initiate a trial of clozapine owing to fear of side-effects such as agranulocytosis.

### Monitor blood levels

Securing drug plasma levels is the only way to know whether treatment failure is due to a pharmacokinetic issue such as rapid metabolism or a cytochrome P450 polymorphism, or simply poor adherence with oral therapy. Likewise, blood levels can alert you to pharmacodynamic abnormalities which occur when treatment response does not correlate with adequate dosing. Blood level monitoring of both FGAs and SGAs can provide the clinician with important information which can guide the treatment plan for patients with treatment-resistant psychosis. This is supported by the recent work of Lopez & Kane as relevant to haloperidol, fluphenazine, perphenazine, risperidone, olanzapine and clozapine.^27^

### Time may not be on your side

As noted above, it takes some patients longer than others to respond to antipsychotic treatments. Granted, it may not be possible to wait several weeks (and certainly not months) in acute settings or when a patient's behaviour is potentially harmful to self or others, but when possible, allowing adequate time for full response may be all that is needed when a patient has exhibited a partial response. The result of impatience is that a second antipsychotic may be prescribed or a single medication may be dosed in an unnecessarily aggressive manner.

### Double-check the diagnosis

It is common practice to rethink the primary diagnosis if the treatment plan appears ineffective. Once pharmacokinetic, pharmacodynamic or time-course failures have been ruled out, the presence of substance misuse or a personality disorder or neurological illness should be considered.

## Antipsychotic polypharmacy

Although a number of published treatment guidelines for schizophrenia are available, some of which conflict with each other, it is clear that clinicians should utilise a monotherapy approach to antipsychotic medication use.^28^ Multiple trials of antipsychotic medications, generally SGAs to include clozapine, are recommended. In fact, divergence from this sequential clinical progression has historically been met with scepticism, caution and outright criticism.^29–36^ It is certainly understandable why this is the case. The literature is replete with evidence supporting the efficacy of monotherapy for schizophrenia. Furthermore, the pitfalls associated with combining antipsychotics are well documented. Increased side-effects, higher medication costs, scant information supporting efficacy, and suboptimal outcomes are all problematic with regard to antipsychotic polypharmacy.^29–36^ So, why the need to even review the topic? The reality is that patients included in research studies are generally those who are able to give consent, exhibit less violence and less impulsivity, have lower rates of chemical dependency, and are less likely to have histories of sequential trials of antipsychotics at documented therapeutic levels.^7,8^ In other words, consistent with much of psychiatry research, they are healthier and not mirror images of the patients seen in clinical practice. Therefore, we believe a strict adherence to a treatment guideline based on highly selective samples does not necessarily translate well to community-based out-patient clinics and in-patient facilities.

We acknowledge that antipsychotic monotherapy is sufficient for the majority of patients with schizophrenia and that adherence to established guidelines should generally occur. Indeed, recent studies support this position. A 2004 study by Suzuki and colleagues revealed that when patients with schizophrenia were switched from multiple antipsychotics to monotherapy, roughly half maintained gains whereas a quarter showed improvements. Another quarter of the sample decompensated.^37^ In a similar study by Essock and colleagues it was found that patients switched to monotherapy maintained gains, but also showed improvement in metabolic effects assumed to be caused by antipsychotic polypharmacy. It should be noted that approximately a third of patients required multiple antipsychotics.^23^ However, some evidence supports the use of antipsychotic polypharmacy. A recent meta-analysis of randomised controlled trials comparing antipsychotic monotherapy and polypharmacy highlighted that polypharmacy may be superior to monotherapy in certain clinical cases.^38^

In addition to achieving adequate D_2_ occupancy, antipsychotic polypharmacy also exploits other receptor-binding properties that could lead to improvement in other schizophrenia symptom clusters. For example, serotonergic, noradrenergic and histaminergic binding theoretically ameliorate depression, anxiety, insomnia, impulsivity and aggression. On the flip side, however, the patient is potentially exposed to adverse side-effects from multiple receptor binding or excessive binding via similar properties shared by antipsychotics (e.g. excessive histaminergic binding leading to daytime sedation or appetite stimulation and weight gain). Consequently, combining antipsychotics should be done rationally based on their binding profiles. One clear example is the need to avoid combining the partial D_2_ agonism of aripiprazole with antipsychotics with full D_2_ antagonism. The binding interference may lead to a worsening of symptoms due to aripiprazole's high affinity for the D_2_ receptor, and the fact that even low doses such as 10 mg achieve 83% D_2_ occupancy, and thus may displace full antagonists.^6^

## High dosing of antipsychotics

Antipsychotic polypharmacy is not the only means of addressing the more complex and treatment-resistant cases of schizophrenia. High-dose monotherapy is a viable option as well. In fact, it has been argued that if the goal is to occupy a greater degree of D_2_ receptors in order to address treatment-resistant positive and aggressive symptoms, high-dose monotherapy is the preferred option when compared with polypharmacy. High-dose monotherapy does, however, come at a greater financial expense and the risk of increased metabolic and other potential treatment-limiting side-effects.^11^

It is impossible to know what dose of a particular antipsychotic is required to achieve the intended outcome. Therefore, the prudent action is to start low within the US Food and Drug Administration (FDA)- and British National Formulary (BNF)-approved guidelines for the particular medication. The medication can be gradually increased outside the FDA-approved dosing window until therapeutic response occurs or the patient develops intolerable side-effects. It is important that informed consent is obtained and treatment rationale is well documented when this occurs. Below we discuss the typical dosing ranges and special considerations for high dosing of the antipsychotics. A more detailed analysis can be found in Stahl & Morrissette's review of the topic.^11^

### Clozapine

Clozapine is typically only recommended after subsequent trials of other antipsychotics have failed. This is primarily owing to its side-effect profile. At typical dosing of 300–450 mg/day, clozapine binds to less than 50% of D_2_ receptors, but as noted earlier, the antipsychotic benefits with this medication can be seen at as low as 20% occupancy.^10^ A meta-analysis by Davis & Chen revealed that patients on high levels of clozapine responded more frequently than those on low levels.^39^ Clozapine can be dosed as high as 900 mg/day, but seizure risk does increase with higher plasma levels, so titration to this dose should be done slowly. Furthermore, due to the diverse binding profile of clozapine, improvement in multiple symptoms clusters is possible.

### Quetiapine

Quetiapine has a relatively weak affinity for D_2_ receptors and often requires high dosing to achieve intended outcomes. Only at the upper range of 400–800 mg/day are the antipsychotic properties of the medication seen. It is generally believed that a dose of 1200 mg/day is no more effective than the typical dosing range and carries greater incidence of metabolic effects; however, clinical practice has shown that 1800 mg/day may be useful in treating violent patients.^2,5,39^

### Olanzapine

Doses of olanzapine between 10 and 20 mg/day equate to 60–80% D_2_ occupancy. Higher doses of 40–60 mg daily appear to be more effective, particularly with aggressive patients and in some forensic settings.^2,32,40,41^ A note of caution is that as plasma levels increase the risks of anticholinergic and metabolic effects also increase.^5,10^

### Risperidone/paliperidone

Risperidone reaches 70–80% of D_2_ occupancy at doses between 2 and 6 mg/day. The risk of EPS is positively correlated with dose. Doses above 8 mg/day are generally not considered beneficial for most patients, but in some, the side-effects may not appear until higher dosages.^5^ As noted previously, even at 10 mg/day only 31% of patients required anti-Parkinsonian medication in the pivotal trials, again providing evidence that a subgroup may both require and tolerate higher dosages and plasma levels.^16^ Risperidone's active metabolite paliperidone has less chance of drug–drug interactions as it is not metabolised by the liver. Similar to risperidone, paliperidone carries increased risk of EPS as the dose increases.^11^

### Ziprasidone

Data support the use of high doses of ziprasidone, particularly in forensic settings at 360 mg/day.^2–5,40,41^ It can be difficult to achieve adequate plasma levels with ziprasidone in out-patient settings as food is required to increase absorption. It has been reported that ziprasidone has historically been under-dosed due to concern about increased agitation and QTc prolongation.

### Aripiprazole

Aripiprazole has a different mechanism of action compared with the ‘first wave’ of SGAs. Contrary to its predecessors, high doses of aripiprazole may not result in increased efficacy in schizophrenia. This is due to its partial agonist properties and high affinity for D_2_ receptors.^11^ Doses of 40 mg/day are associated with 96.8% D_2_ occupancy, so further increases will not have an impact on D_2_ neurotransmission to any considerable extent.

### Asenapine, iloperidone, and lurasidone

Asenapine, iloperidone, and lurasidone are newer atypical antipsychotics. Consequently, there is limited information that supports their use in high doses. Although doses of asenapine of 30–40 mg/day may be effective for some treatment-resistant cases, there are virtually no data supporting use at these higher doses, and the buccal absorption of asenapine declines significantly for each 5 mg increase in the dose. As with asenapine, there are limited to no data supporting the use of iloperidone at high doses. One treatment-limiting issue with iloperidone is orthostatic hypotension. Lurasidone is approved up to 160 mg/day for schizophrenia, but higher dosages have not been studied for efficacy, only for safety (e.g. thorough QT studies up to 600 mg). Similar to ziprasidone, lurasidone should be taken with food to increase absorption.^11^

## Summary

Schizophrenia is a relatively common psychiatric disorder but it is often difficult to treat. Although antipsychotic monotherapy at standard dosing levels is sufficient for the majority of patients, a subset will require ‘unconventional’ approaches such as antipsychotic polypharmacy and higher than normal dosing. If done cautiously and rationally, these approaches can provide much-needed benefit for those most in need of relief.
